# Supplementary material for: Health care providers’ awareness of breastfeeding practice recommendations during COVID-19 pandemic and associated factors in Northwest Ethiopia, 2021: A multicenter study
Source: PLoS One. 2021 Dec 10;16(12):e0260762. doi: 10.1371/journal.pone.0260762 (PMC8664227; doi:10.1371/journal.pone.0260762)
Supplement: S1 File — (DOCX) [file pone.0260762.s001.docx]

| **Questionnaire** | | |
| --- | --- | --- |
| **A** | **Socio-demographic related questions** | |
| **Sr.N.** | **Questions** | **Response** |
| 01 | Your age in years | _____________ |
| 02 | Your sex | 1. Male 2. Female |
| 03 | Current marital status | 1. Single 2. Married |
| 04 | Do you have smart phone and or computer? | 1. No 2. Yes |
| 05 | Average monthly income | _______in Ethiopian Birr |

Title**:** Health care providers’ awareness of breastfeeding practice recommendations during COVID-19 pandemic and associated factors in Northwest Ethiopia, 2021

| **Questionnaire** | | | |
| --- | --- | --- | --- |
| **B** | **Work place and profession related characteristics** | | |
| **Sr.N.** | **Questions** | **Response** | |
| 01 | Professional category | 1. Diploma Midwifery 2. BSc Midwifery 3. MSc Midwifery 4. Other | |
| 02 | Hospital type | 1. Primary hospital 2. General hospital 3. Tertiary hospital | |
| 03 | Facility location | 1. Urban 2. Semi-urban | |
| 04 | Work experience in years | _____________ | |
| 05 | Do you think that COVID-19 is dangerous? | 1. Yes 2. No | |
| 06 | Did you take COVID-19 online training prepared by Ethiopian public health institute? | 1. Yes 2. No | |
| 07 | Do you follow the WHO/CDC COVID-19 guideline updates? | 1. Yes 2. No | |
| 08 | Have you ever received training on Essential newborn care? | 1. Yes 2. No | |
| 09 | Do you use internet as a source of information on COVID-19? | 1. Yes 2. No | |
| 10 | Do you believe that COVID-19 preventive measures are effective? | 1. Yes 2. No | |
| **Questionnaire** | | | |
| **C** | **Awareness to the WHO breastfeeding practice recommendation during COVID-19 pandemic in suspected or confirmed cases** | | |
| **Sr.N.** | **Questions** | | **Response** |
| 01 | Early skin to skin contact is recommended | | 1. Yes 2. No |
| 02 | The mother should be told that the benefit of breast feeding outweighs the potential risks of COVID-19 transmission | | 1. Yes 2. No |
| 03 | Human milk contains viable SARS-CoV-2 | | 1. Yes 2. No |
| 04 | Umbilical cord should be clamped as usual | | 1. Yes 2. No |
| 05 | Breastfeeding Should be initiated within one hour after delivery | | 1. Yes 2. No |
| 06 | Direct breastfeeding is recommended | | 1. Yes 2. No |
| 07 | A mother with COVID-19 can give expressed breast milk if she is too unwell to directly breastfeed | | 1. Yes 2. No |
| 08 | A COVID-19 suspected or confirmed mother should take routine precaution measures | | 1. Yes 2. No |
| 09 | Donor human milk or wet nursing can be used if a woman is severely infected with COVID-19 | | 1. Yes 2. No |
| 10 | Rooming-in is recommended? | | 1. Yes 2. No |
