# Supplementary material for: Health care providers’ awareness of breastfeeding practice recommendations during COVID-19 pandemic and associated factors in Northwest Ethiopia, 2021: A multicenter study
Source: PLoS One. 2021 Dec 10;16(12):e0260762. doi: 10.1371/journal.pone.0260762 (PMC8664227; doi:10.1371/journal.pone.0260762)
Supplement: Attachment — Submitted filename: Response to Reviewers.docx [file pone.0260762.s003.docx]

**November 15, 2021**

**Manuscript ID:** PONE-D-21-32452 **(**Health care providers’ awareness of breastfeeding practice recommendations during COVID-19 pandemic and associated factors in Northwest Ethiopia, 2021: a multicenter study)

**Response to Reviewers**

First of all, we would like to thank the reviewers and the editor for their constructive comments and valuable improvements to the manuscript. We have incorporated all the suggestions and comments. The changes made during revision have been shown with tracked changes and a point-by-point response-please see below.

**Response to Reviewer(s) comments**

**Reviewer #1**

| S.N | Comments | Responses |
| --- | --- | --- |
| 01 | Regarding the data availability, authors agreed it’s available, is it that SPSS file at the end of document? | Thank you for understanding, the SPSS data is available at the end of the submission. |
| 02 | Table 1 title is mentioned twice, please check. | Thank you, dear reviewer. It has been corrected in the revised manuscript. |
| 03 | How p-value of 0.2 is set for multivariable regression? | Thank you dear reviewer for your concern. Please accept our apologies, it was typing error. The basis was to take all variables having a p-value of <0.25 to set for the multivariable regression. However, it was written < 0.2 and it was typing error. We did based on the assumption to include all variables having a p-value of <0.25 as evidenced by existing literature (<http://dx.doi.org/10.21037/atm.2016.02.15>, doi: 10.1186/1751-0473-3-17, doi: 10.1186/1751-0473-3-17). For this study, we used a p-value of 0.25 assumption to proceed to the multivariable logistic regression, however, all variables which fulfill the chi-square assumption had a p-value of ≤ 0.2. The principle we used has been corrected in the revised manuscript, in which even if all the variables had a p-value of ≤ 0.2, the standing assumption was to include variables having a p-value <0.25. Thank you once again for raising this important question. |

**Reviewer #2**

| S.N | Comments | Responses |
| --- | --- | --- |
| 01 | Technical sections such as Materials & Methods section needs to expanded for the reader to completely grasp the concept, For example, Clear definition of what an HCP (Health Care Professional) is within the confines of the study needs to be stated, there is a need for explicitly stating why Gondar Province Hospitals were selected as a study setting and why was it only restricted to Government Hospitals - the same needs to be Substantiated with a Rationale, Also For computation of sample size justification for using 50% as provider awareness needs to be clearly stated. | Thank you, dear reviewer. The HCP for this study has been put in the study population section. Gondar province is a wider area that contains almost 5 zones (south, central, north, west, and Gondar city zones). All hospitals in these zones were considered. It was possible to include other areas, but since there was no funding, it was not possible to include more than these areas (i.e. feasibility issue). Besides, we included only governmental hospitals because there were only two private hospitals in the province and based on the available evidence, most of the staff in the private hospitals are governmental hospital employees too as a per time workers. In this case, there might be a probability of including one person more than once. This may affect the result of our study. Regarding the sample size calculation, since there were no similar studies in Ethiopia as the authors’ deep review and to get a maximum number of samples we used 50%. |
| 02 | As per the guidelines enlisted, the Tables within the article should be included directly after the paragraph in which they are first cited. | Thank you, dear reviewer. During submission, the list of tables should be put at the end of the manuscript or submitted separately ((PLOS ONE submission guideline). However, each table should be cited immediately after the paragraph in sequential order. After the manuscript gets published, the full table will be placed in the first cited paragraph. |
| 03 | The authors need to explicitly state the cut off one considered being aware or unaware that were used for computation and drawing inference to improve the understanding of the reader. | Thank you, dear reviewer. We tried to make it clear in the revised manuscript and shown with track changes. |
| 04 | The Authors to Cross check the Gender disaggregation stated in the Result section and the Appendix Table | Thank you dear reviewer. It has been corrected in the revised manuscript. |
| 05 | Adherence to the sequence: Acknowledgments, References, Supporting information captions (if applicable) is required per the guidelines | Thank you, you are we have looked at the sequence and it is based on your kind suggestion. We tried to put the sequence based on the guideline and previously published papers. There is no hard and fast rule regarding this and if there is a problem with the sequence, the journal will guide and communicate with us. We really appreciate your deep reading and special attention to each part of the manuscript. |
| 06 | Revision of language and formatting in a couple of places is required, including cross checking of the numbering of the Tables | Thank you dear reviewer. We thoroughly revised the whole manuscript for the English language and cross-checking the numbers of each table. |
| 07 | The additional information under the tables are to be included in the form of a footnote | Thank you, dear reviewer. We tried to put the notes and abbreviations under tables based on previously published papers and the journal guideline. |
| 08 | There is a need to recheck and edit the References to meet the Vancouver format guidelines | Thank you, dear reviewer. We tried to check the reference based on the Vancouver referencing style. However, since most of the references are guidelines and websites, it might not completely fulfil the style ((scarce of related published articles). |
| 09 | The limitation section, needs to further highlight whether there are any potential bias, confounding variables that would potentially have an impact on the findings and inferences drawn, if any | Thank you, dear reviewer, for your insightful comment. We have put list the potential limitations in the revised manuscript. However, there were no confounding variables since we have checked the multicollinearity assumptions and the variance inflation factor (VIF) was < 10 and we have incorporated it in the data analysis section in the revised manuscript. Thank you once again for reaching out to this important comment for the improvement of our manuscript. |

**Reviewer #3**

| S.N | Comments | Responses |
| --- | --- | --- |
| 01 | Overall, this manuscript may be useful to know what factors contribute to health care providers' awareness of breastfeeding practice recommendations. This research contributes to the body of knowledge and database. | Thank you dear reviewer for your support and positive insight. |
| 02 | In abstract section, the data collection duration need to be corrected. | Thank you, dear reviewer. It was typing error and corrected in the revised manuscript. |
| 03 | Observations that the breastfeeding practice recommendations are being discussed for mothers suspected of or diagnosed with Covid-19 came very late in the process. It should be emphasized right from the start, starting with the objectives. | Thank you, dear reviewer, for your perceptive comment. The main objective of the study was how many of the healthcare providers are aware of breastfeeding practice recommendations during COVID-19 and what factors was responsible for the increased or decreased, thereby putting important recommendations. However, we didn’t make any observation for the breastfeeding practice by ourselves. |
| 04 | Under data processing and analysis, it is stated that those variables with p value 0.2 were included in logistic regression. What is the basis for taking such value? This raises a concern for overall regression analysis. This needs to be relooked. | Thank you dear reviewer for your concern. Please accept our apologies, it was typing error. The basis was to take all variables having a p-value of <0.25 to set for the multivariable regression. However, it was written < 0.2 and it was typing error. We did based on the assumption to include all variables having a p-value of <0.25 as evidenced by existing literature (<http://dx.doi.org/10.21037/atm.2016.02.15>, doi: 10.1186/1751-0473-3-17, doi: 10.1186/1751-0473-3-17). For this study, we used a p-value of 0.25 assumption to proceed to the multivariable logistic regression, however, all variables which fulfill the chi-square assumption had a p-value of ≤ 0.2. The principle we used has been corrected in the revised manuscript, in which even if all the variables had a p-value of ≤ 0.2, the standing assumption was to include variables having a p-value <0.25. Thank you once again for raising this important question. |
| 05 | Under the results section, under factors associated- it was mentioned that good or bad awareness. This is a wrong use of terminologies as there is nothing like good or bad. But instead, there could be certain factors which were associated with more likelihood of awareness. The results may be rewritten for better appropriate use of terminology. | Thank you, dear reviewer. We fully accept the comment and corrected in the revised manuscript. |
| 06 | The manuscript must be extensively revised for language and grammar | Thank you, dear reviewer. We thoroughly revised the manuscript for English language and grammar in the revised manuscript. |
| 07 | We understand that there is paucity of evidence in this area and this limited the discussion in comparison with other studies. However, the discussion currently is giving results more quantitatively rather than a perspective that can be a little qualitative. May be a discussion around what the guidelines say and what is observed in the current study could be talked a little more. Apart from just the predictors, it may also be good to understand awareness around which indicators based on the questions used in the questionnaire is low and needs more attention. This will tell the readers what areas need more attention for creating awareness among health care providers. | Thank you, dear reviewer, we accept it. It is very good recommendation and we tried to do that in the revised manuscript immediately after discussing the proportion of awareness. Thank you once again for helping us to improve our manuscript. |
